# Supplementary material for: Causal Relationship between Aging and Anorexia Nervosa: A White-Matter-Microstructure-Mediated Mendelian Randomization Analysis
Source: Biomedicines. 2024 Aug 16;12(8):1874. doi: 10.3390/biomedicines12081874 (PMC11351342; doi:10.3390/biomedicines12081874)
Supplement: Supplementary file 1 [file biomedicines-12-01874-s001.zip › Supplementary Figure/Figure S4/Singe SNP plot of MR analysis for TL to ubm-b-1633.pdf]

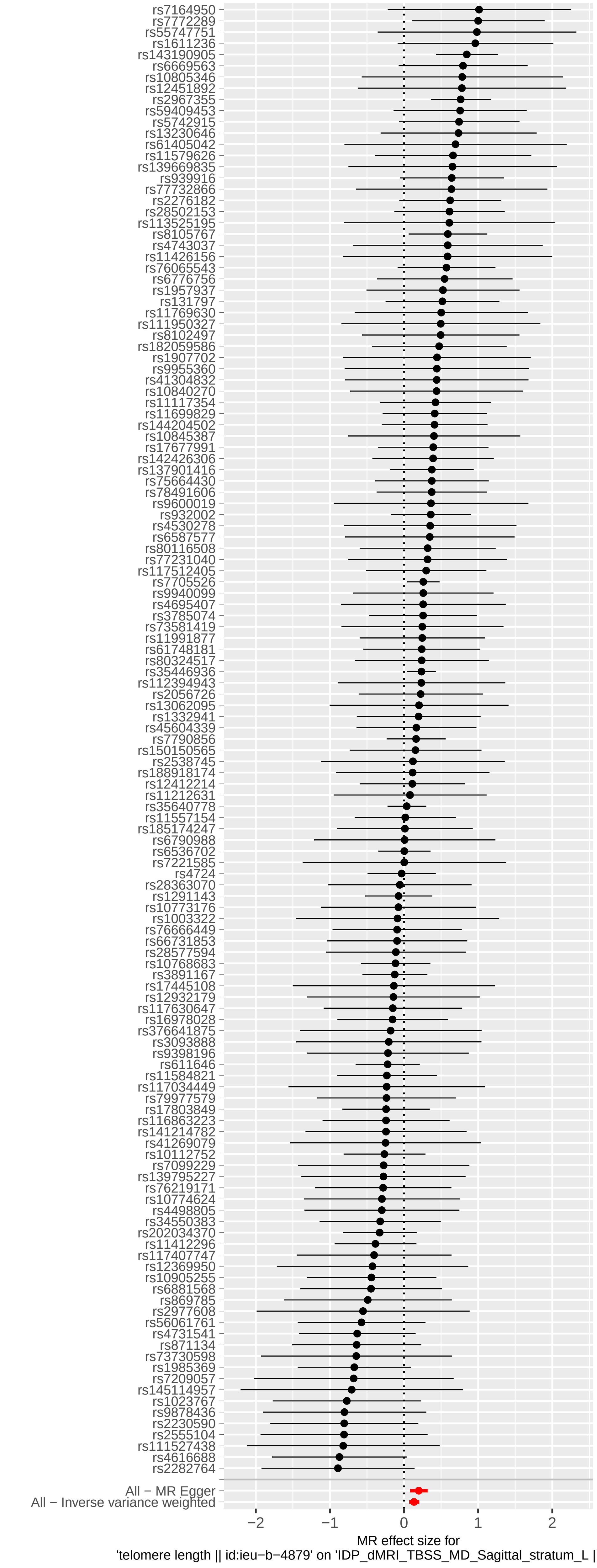
rs7164950
rs7772289
rs55747751
rs1611236
rs143190905
rs6669563
rs10805346
rs12451892
rs2967355
rs59409453
rs5742915
rs13230646
rs61405042
rs11579626
rs139669835
rs939916
rs77732866
rs2276182
rs28502153
rs113525195
rs8105767
rs4743037
rs11426156
rs76065543
rs6776756
rs1957937
rs131797
rs11769630
rs111950327
rs8102497
rs182059586
rs1907702
rs9955360
rs41304832
rs10840270
rs11117354
rs11699829
rs144204502
rs10845387
rs17677991
rs142426306
rs137901416
rs75664430
rs78491606
rs9600019
rs932002
rs4530278
rs6587577
rs80116508
rs77231040
rs117512405
rs7705526
rs9940099
rs4695407
rs3785074
rs73581419
rs11991877
rs61748181
rs80324517
rs35446936
rs112394943
rs2056726
rs13062095
rs1332941
rs45604339
rs7790856
rs150150565
rs2538745
rs188918174
rs12412214
rs11212631
rs35640778
rs11557154
rs185174247
rs6790988
rs6536702
rs7221585
rs4724
rs28363070
rs1291143
rs10773176
rs1003322
rs76666449
rs66731853
rs28577594
rs10768683
rs3891167
rs17445108
rs12932179
rs117630647
rs16978028
rs376641875
rs3093888
rs9398196
rs611646
rs11584821
rs117034449
rs79977579
rs17803849
rs116863223
rs141214782
rs41269079
rs10112752
rs7099229
rs139795227
rs76219171
rs10774624
rs4498805
rs34550383
rs202034370
rs11412296
rs117407747
rs12369950
rs10905255
rs6881568
rs869785
rs2977608
rs56061761
rs4731541
rs871134
rs73730598
rs1985369
rs7209057
rs145114957
rs1023767
rs9878436
rs2230590
rs2555104
rs111527438
rs4616688
rs2282764
All − MR Egger
All − Inverse variance weighted
−2
−1
0
1
2
MR effect size for
'telomere length || id:ieu−b−4879' on 'IDP_dMRI_TBSS_MD_Sagittal_stratum_L ||
